# How Does Hands-On Making Attitude Predict Epistemic Curiosity and Science, Technology, Engineering, and Mathematics Career Interests? Evidence From an International Exhibition of Young Inventors

**DOI:** 10.3389/fpsyg.2022.859179

**Published:** 2022-05-20

**Authors:** Yuting Cui, Jon-Chao Hong, Chi-Ruei Tsai, Jian-Hong Ye

**Affiliations:** ^1^Faculty of Education, Beijing Normal University, Beijing, China; ^2^Department of Industrial Education, National Taiwan Normal University, Taipei, Taiwan; ^3^Institute for Research Excellence in Learning Sciences, National Taiwan Normal University, Taipei, Taiwan; ^4^International Master Program in STEM Education, National Pingtung University, Pingtung, Taiwan

**Keywords:** STEM, epistemic curiosity, invention exhibition, hands-on making attitude, STEM career interest

## Abstract

Whether the hands-on experience of creating inventions can promote Students’ interest in pursuing a science, technology, engineering, and mathematics (STEM) career has not been extensively studied. In a quantitative study, we drew on the attitude-behavior-outcome framework to explore the correlates between hands-on making attitude, epistemic curiosities, and career interest. This study targeted students who joined the selection competition for participating in the International Exhibition of Young Inventors (IEYI) in Taiwan. The objective of the invention exhibition is to encourage young students to make innovative projects by applying STEM knowledge and collaborative design. We collected 220 valid data from participants in the 2021 Taiwan IEYI selection competition and conducted a confirmatory factor analysis and structural equation modeling to test the hypotheses. Results indicated that: (1) hands-on making attitude was positively related to two types of epistemic curiosity; (2) interest-type epistemic curiosity (IEC) and deprivation-type epistemic curiosity (DEC) were positively associated with STEM career interest; additionally, DEC had a higher coefficient on STEM career interest than IEC; (3) both types of EC had a mediating role between hands-on making attitude and STEM career interest. It is expected that encouraging students to participate in invention exhibition competitions can raise both types of EC and increase their interest in pursuing STEM careers.

## Introduction

In order to ensure that young students can meet the growing demand for creativity in science, technology, engineering, and mathematics (STEM) fields, educators must help them learn to think outside the box (Hardy et al., 2017), and learn new procedural and declarative knowledge to creatively design projects (Nazzal and Kaufman, 2020). Higher levels of depth capability to learn new knowledge result in a greater impact on invention (Kok et al., 2019). Hands-on attitude and open-ended exploration are integral components of good STEM design (Wu et al., 2014; Hu et al., 2020), which implies that STEM creative exploration should play a role in activating students’ curiosity (Hong et al., 2016, 2019). That is, epistemic curiosity is always accompanied by motivation to solve problems about one’s surrounding environment (Buyalskaya and Camerer, 2020), indicating that project design with STEM knowledge may impact Students’ entrance into the STEM career pipeline (Birenbaum et al., 2021). However, few studies have focused on exploring the correlates between hands-on making attitude, epistemic curiosity, and career interest. Thus, the present study aimed to explore their correlations.

Which factors drive creative activity? According to the Attitude-Behavior-Outcome (ABO) model, attitudes are robust predictors of appraisal of behavior and decision making (Ajzen and Fishbein, 1980; Nabi et al., 2006). Epistemic curiosity (EC) refers to one’s desire to acquire new knowledge, and comprises two different types after engaging in activities. On one hand, it can arouse positive feelings of intellectual interest, which is known as Interest type EC (IEC). On the other hand, it can reduce the undesirable condition of uncertainty that is associated with being deprived of information, which is referred to as Deprived type EC (DEC). As an example, scientists perform behaviors to seek knowledge from context-specific information provided by contextual situations to alleviate the pressure from knowledge gaps that can reduce uncertainty about specific unknowns, and consequently achieve their goals. Moreover, genuine interest in a STEM career relies on individuals’ major or experience, which influences their decisions to choose a career (Kim and Beier, 2020). However, few studies have investigated STEM experience in a contest involving invention model making and competitions in which Students’ hands-on making attitudes activate their epistemic curiosity related to their career interest. Thus, drawing on ABO, the present study formed a research framework to explore the correlates between hands-on making attitude, IEC and DEC, and career interest. It is expected that the study results can be applied in Taiwan educational settings which focus on Confucian culture.

## Theoretical Background

### Science, Technology, Engineering, and Mathematics Education

STEM education has become an important part of curricula in educational systems around the world (e.g., Bagiati and Evangelou, 2015; Al Salami et al., 2017; Margot and Kettler, 2019), and in particular, has seen successful implementation in a number of Western countries including the United States and Australia (Lee et al., 2019). In a special issue on STEM education in 2019, Le et al. (2021) called for further investigation of how STEM education is implemented in Asian schools. Since then, the difficulties of implementing STEM education have received increasing attention, and there has been a search for effective educational approaches and curricula (Le et al., 2021). It has been argued that STEM narratives of progress, competition, and innovation have obscured some of the issues that students must face on a daily basis, including urgent ecological, ethical, and social justice issues (Yanez et al., 2019). Thus, an approach should be practiced with these critical principles—production pedagogy—in mind when applying STEM knowledge in a competition. In the process of producing projects, students engage in critical discussion and make alternative models which generate new perspectives on how they might “do” differently and innovatively. In line with this, a focus on “doing” to learn STEM to design products for an International Exhibition of Young Inventors (IEYI) competition was emphasized in this study.

### Hands-On Making Attitude

Attitudes have been defined as an individual’s cognitive preferences and behavioral predispositions toward objects, which result in either favorable or unfavorable evaluations of certain stimuli that reflect that individual’s tendency (Eagly and Chaiken, 1993). This psychological tendency conveys the individual’s evolution of referents (Augoustinos et al., 2014) and their resulting behavioral intentions (Ajzen, 2001), Moreover, hands-on learning has been defined as any instructional approach that involves students in actively manipulating objects so as to develop their knowledge or understanding (Haury and Rillero, 1994). Activity-centered learning is used synonymously with hands-on making, including manipulative activities and practical activities with hands-on activities (Ateş and Eryilmaz, 2011). A previous study suggested that people’s attitudes are defined as a stable trait that is formed *a priori* and is activated unconsciously in response to either the internal or external stimuli provided by an activity (Serenko and Turel, 2019). However, students have reported hindering attitudes toward doing innovative research because they may consider that innovative activities are time-consuming, and they may face difficulties completing the activities due to their lack of knowledge (AlGhamdi et al., 2014). In line with this, the role of participants’ hands-on making attitudes in designing a project for IEYI was considered in this study.

### Epistemic Curiosity

Loewenstein’s (1994) information gap theory of curiosity was recently extended by Litman and Jimerson (2004) and Litman (2005) to include both the interest (I-type) dimension, which involves the acquisition of novel information which can generate positive feelings of interest, and the deprivation (D-type) dimension, which is related with minimizing uncertainty and eliminating undesirable states of ignorance. Epistemic curiosity is described as the individual’s “desire to know” novel knowledge that shrinks the discrepancy (knowledge-gap) of the “need to know” between known and desired information (Litman et al., 2005). Previous studies have indicated that curiosity has a central function in hands-on exploration and intellectual behavior (Murayama et al., 2019), and would directly predict positive effort beliefs and goal orientation (Grossnickle, 2016). Epistemic curiosity (EC) is a distinctive human tendency to drive cognitive inquisition. IEYI is a material- and activity-centered project-making STEM competition which emphasizes hands-on as well as minds-on activities. Hands-on making projects require students to acquire knowledge and discuss with peers and/or instructors. In making an invention, students have to co-produce knowledge which interconnects scientific and technical knowledge (Eaton et al., 2021) and in ways of practicing epistemic curiosities (Birenbaum et al., 2021). This suggests that students involved in design invention drive themselves to learn more knowledge, for example, to know how different sensors can work. Thus, the roles that both types of epistemic curiosity play in making inventions were explored in this study.

### Career Interest

STEM-centered learning activities comprise both activities carried out in school and out-of-school (OOS) STEM programs (Kong et al., 2014). OOS STEM programs designed for young teenagers emerged in association with the development of the competencies and the corresponding confidence considered suitable for a STEM career (Gagnon and Sandoval, 2020). An example is a program comprising semi-structured courses which explored how students could continue to engage across a range of STEM career pathways. The results suggested that specifically focusing on STEM programs for young people could encourage them to pursue a STEM career in the future (Beier et al., 2019). Moreover, Kang et al. (2019) suggested that OOS programs may provide the necessary context for developing young people’s socio-emotional and motivational skills along with their STEM career aspirations and career determinism. However, although STEM education has been found to be able to motivate students to study STEM and to pursue future STEM careers (Lee et al., 2019; Margot and Kettler, 2019), how an event such as IEYI may develop adolescent participants’ career interest in STEM is still unknown. Thus, participants’ career interest was explored in this study.

## Research Hypotheses and Model

### Hands-On Making Attitude and Epistemic Curiosity

IEC refers to the “desire to know” know-how, and results in positive feelings of intellectual interest in and enjoyment of cognitive tasks which require effort, while DEC refers to the “need to know” in the motivation to reduce undesirable states of informational deprivation in cognitive tasks (Strobel, 2014). In hands-on making contests, project design can activate Students’ curiosity to produce different kinds of artifacts (Mohr-Schroeder et al., 2014). Moreover, most people are not even aware of the existence of attitude and its implicit impact on their behavior, and so they often refer to their automatically driven actions (Serenko, 2022). When students experience pleasure in cognitive activities, positive affect will promote high levels of curiosity, which is more conducive to problem solving and exploratory behaviors (e.g., Hong et al., 2016). Evidence supports the link between curiosity and attitude of hands-on making, underlying which is a high degree of uncertainty, novelty preference, and dynamic complexity (Jirout and Klahr, 2012). Accordingly, how hands-on making attitude related to participants’ two types of epistemic curiosity when working collaboratively on STEM making and design of inventions in competition groups was hypothesized as follows:

H1: Hands-on making attitude is significantly related to IEC.
H2: Hands-on making attitude is significantly related to DEC.

### Epistemic Curiosity and Science, Technology, Engineering, and Mathematics Career Interest

IEC orients individuals toward a carefree form of intellectual exploration (Lauriola et al., 2015), relates with acquiring knowledge purely for the intrinsic pleasure, and is associated with “drive to know” (Litman, 2008). In contrast, DEC reflects a state of dissatisfaction with a specific problem and is conceptualized as a “need to know” with moderately unpleasant feelings (Litman, 2008; Subaşı, 2019). It is correlated with pervasively negative emotions such as depression, anxiety, and burnout (Litman, 2008; Kashdan et al., 2020). Nevertheless, some empirical studies have indicated that DEC orients individuals to have positive relationships with performance achievement, intrinsic motivation, self-growth, stress tolerance, and perseverance to master goal orientation (Litman et al., 2010; Kashdan et al., 2018). It also influences one’s career optimization and professional life (Malcom et al., 2020). However, many studies have taken epistemic curiosity as one variable, with few separately exploring whether the different types of EC (drive to know and need to know) play different roles in career interest (Tang and Salmela-Aro, 2021). Furthermore, few studies have connected epistemic curiosity to hands-on STEM activities, an important pathway to develop young Students’ team competitiveness and STEM career interest (Wright and Walton, 2003; Mussel, 2013). To understand how both types of EC relate to participants’ future career interest was hypothesized as follows:

H3: I-type EC is positively correlated to STEM career interest.
H4: D-type EC is positively correlated to STEM career interest.

### Hands-On Making Attitude and Science, Technology, Engineering, and Mathematics Career Interest

Epistemic curiosity is driven by positive attitudes. Individuals who are placed at a high level of EC will probably be highly motivated and engaged in learning, and tend to frequently exhibit explorative behaviors in choosing scientist and inventor careers (Birenbaum et al., 2021). Moreover, in comparison with conventional settings, the essence of the IEYI contest is the process of hands-on creation with STEM knowledge. Such a competitive environment fosters more interest and positive attitudes toward invention projects (Huang et al., 2016). Accordingly, their hands-on making attitude supports the development of their behavioral intentions, thus leading to actual career intention (Serenko, 2022). In this study, considerable attention to the Attitude-Behavior-Context (ABC) model (Guagnano et al., 1995) links to examining participants’ attitudes in the specific context of invention design, in light of epistemic curiosity, which ultimately influences their career interests. However, few studies have explored whether hands-on making attitude can directly enhance Students’ intention to participate in STEM careers. Hence, we proposed the following hypothesis:

H5: Hands-on making attitude is significantly related to STEM career interest mediated by two types of epistemic curiosity.

### Research Model

**FIGURE 1:**
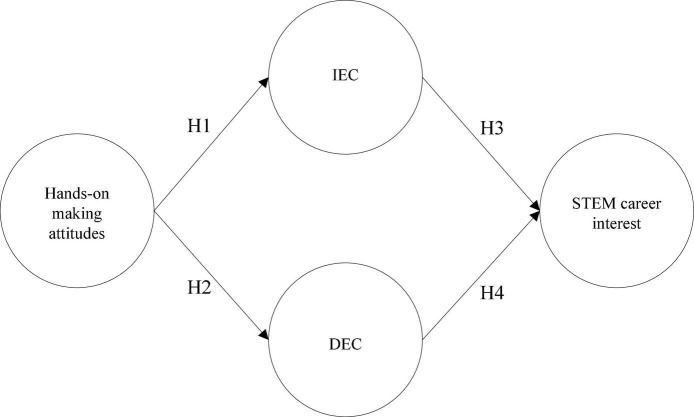
Research model.

This study employed the ABO framework, which has been widely used to understand behavior and outcomes in various settings (e.g., Hansen, 2008; Ashnai et al., 2016). The pursuit of STEM-related career interests could be the outcome regarded as career decision-making in which students evaluate the person-vocation fit of their career interest (Kim and Beier, 2020). Hence, we utilized these multiple links in our models (presented Figure 1) to confirm the influences of hands-on making attitudes which activated two inquisitive behaviors of Students’ epistemic curiosity then predicted the outcomes of their STEM career pursuits in a real hands-on making contest.

## Materials and Methods

### Research Setting

IEYI is a science and technology contest with the designated goal of providing students with the design knowledge and skills needed for their future careers, and of developing Students’ core competences such as problem solving, critical thinking, and communication skills. Participants should prepare a complete description of the work and the outstanding information related to their work. Professional judges evaluate the inventions based on the principles of creativity (e.g., innovative function; innovative mechanism; application of scientific nature), marketability benefits (e.g., market demand; social contribution; appearance and exquisiteness) and operability (e.g., operation, constitutive property, and overall integration). Therefore, participating team members need to implicitly learn and use scientific knowledge, mathematical principles, and creative thinking to design their prototypes, as well as technical knowledge, engineering ability, and practical skills to create projects. Also, participants should decorate and articulate their artworks with creative forms and have intelligible expression when presenting their projects. Engaging in this task-specific activity certainly poses cognitive challenges for youth, as they must quickly and accurately come up with solutions to turn their imaginations into concrete innovative products.

### Participants and Research Procedure

This study used purposive sampling. The participants were competitors from vocational high schools in Taiwan who participated in the 2021 IEYI Taiwan Competition held on March 16, 2021, and freely signed up in teams of up to three students. The questionnaire data were filled out and collected anonymously while taking time out of the competition. Participants who did not wish to complete the questionnaire could withdraw from the study without any repercussions.

After the competition, 236 questionnaires were collected. In a preliminary review, questionnaires with missing responses or answers that were all the same were excluded; the valid questionnaires numbered 213, with an effective response rate of 91.5%. The analysis sample consisted of 128 (60.71%) males and 85 (40.4%) females.

### Questionnaire

The questionnaires were designed by referring to prior studies and relevant theories to assure their face validity (Hardesty and Bearden, 2004). The original items were then translated into Chinese and were reviewed by domain experts for both accuracy and intelligibility. A 5-point Likert scale was used, where 1 = *very slightly* or *not at all* and 5 = *extremely*.

Hands-on making attitude (HMA): Three attitude components: affect, behavior, and cognition (knowledge and beliefs) were identified by Breckler (1984). In addition, according to the Expected value theory, attitude has evolved as an integrated framework of needs, expectations and values, and could be used as an explanation of Students’ multidimensional attitudes toward hands-on making (Zhang et al., 2021). Considering that attitude definition differs depending on the culture or domain (Vogel and Wänke, 2016), the construct items of hands-on making attitude were adapted from Hong et al.’s (2021) study, which included affect and behavior and were developed to assess students’ tendency to engage in hands-on problem-solving. The scale has seven items such as “I like to assemble things following the manual instructions,” and “When an electrical appliance breaks down (e.g., an electric fan), I will try to repair it by myself first.”

Epistemic curiosity: Two different epistemic curiosity scales were composed to assess the IEC and DEC components adapted from Litman and Spielberger (2003). Each has six items. For I-type EC, example items are, “The more complex the invention, the more I enjoy exploring its innovativeness,” and “When I find a novel invention, I will explore its features and functions by browsing all kinds of information.” For DEC, exemplary items are, “When I’m working on a creative project, I will continue to explore the causes of other problems that arise after one problem,” and “When I encounter challenges in inventing, I will estimate and probe ways to solve them.”

STEM career interest (SCI): For this scale, the Career Interest Questionnaire (CIQ) (Tyler-Wood et al., 2010) was compiled. This scale consists of seven items such as “I will enjoy a career in science” and “When I graduate, I will specialize in the field required for a career in science or engineering.”

## Results

### Item Analysis

In order to ensure the suitability of items in each construct, firstly, items with factor loadings less than 0.5 were deleted. Next, a first-order confirmatory factor analysis (CFA) was conducted to check the internal validity of each item through excluding items which showed the highest residuals for each construct until they reached the recommended threshold (Hair et al., 2019b). Accordingly, the *x*^2^*/df* value should remain below 5, the RMSEA is in the range of 0.05–0.10, and the expected of GFI and AGFI values should be above 0.80. As the results of executive Model Fit Statistics are shown in Table 1, the deletions in this study were that HMA was reduced from seven to four items, the IEC and DEC items were both reduced from six to four, and the SCI items were reduced from seven to six.

**TABLE 1:** Results of first-order confirmatory factor analysis—model fit measures.

Index	Threshold	Hands-on making attitude	I-type EC	D-type EC	STEM career interest
*x* ^2^ */df*	<5	2.421	1.419	1.959	2.098
RMSEA	<0.10	0.081	0.044	0.066	0.071
GFI	>0.8	0.978	0.993	0.982	0.971
AGFI	>0.8	0.934	0.967	0.947	0.933
FL	>0.5	0.570∼0.831	0.753∼0.838	0.681∼0.841	0.624∼0.878
*t*-value	>3	15.563∼22.368	17.151∼22.150	16.828∼17.813	14.835∼18.148

To examine the external validity of each item, we performed an independent sample *t*-test. The top 27% of the scale scores were categorized as high and the bottom 27% as low. According to Cor (2016), the resultant value should be above 3 to be considered as statistically significant. In this study, the *t*-value was higher than 13.59 (*p* < 0.001^***^), demonstrating that the model had good discriminant and external validity, and could be used for different samples in different situations (Green and Salkind, 2004).

### Reliability and Validity Analyses

Questionnaire reliability was assessed by Cronbach’s α and composite reliability (CR). The Cronbach’s α value should be above 0.7 (Tavakol and Dennick, 2011) and CR should exceed the 0.7 threshold (Lleo et al., 2021). As shown in Table 2, the Cronbach’s α values above 0.83 reveal that those constructs have good internal consistency; the CR values above 0.70 indicate that they have acceptable external consistency. Further, the convergent validity of the constructs was verified by the AVE and factor loading, where the values should exceed 0.5 (Lleo et al., 2021). Table 2 shows that the FL and AVE of all variables were above 0.6, signifying that there is acceptable convergent validity for all constructs.

**TABLE 2:** Construct reliability and validity analysis (*n* = 213).

Constructs	*M*	*SD*	α	CR	FL	AVE
Hands-on making attitude	3.824	0.756	0.826	0.894	0.757	0.628
I-type EC	4.358	0.631	0.880	0.882	0.808	0.653
D-type EC	4.272	0.671	0.864	0.894	0.787	0.628
STEM career interest	4.246	0.70	0.903	0.904	0.767	0.613

To ensure construct discriminant validity (i.e., the difference between two constructs), it is recommended that the correlation coefficient between two constructs be less than the square root of the AVE of each construct (Awang et al., 2015). As can be seen in Table 3, the square root of the AVE of each construct exceeded the absolute value of the correlation coefficients between constructs. Thus, the questionnaire had good construct discriminative validity.

**TABLE 3:** Construct discriminative validity analysis (*n* = 213).

Constructs	1	2	3	4
Hands-on making attitude	**0.792**			
I-Type EC	0.459	**0.808**		
D-Type EC	0.592	0.776	**0.792**	
STEM career interest	0.487	0.679	0.712	**0.783**

*Bold values on the diagonal are the square roots of AVE. To establish the discriminative validity, the value should be greater than the inter-construct correlations.*

### The Structural Model Fit Analysis

In the study, we used AMOS 20.0 to analyze the model fit. As a large number of fit statistics consider different aspects of the fit, Thompson (2000) suggested that researchers should report multiple fit statistics in structural equation modeling studies. According to the absolute fit measures, the recommended values including *x*^2^*/df* should be less than 5, RMSEA should be less than 0.1, and GFI and AGFI should be greater than 0.80 (Hair et al., 2019a). As for the incremental fit measures, the fair fit indicators include NFI, TLI, CFI, IFI, and RFI which should all be larger than 0.8 (Hair et al., 2019a). In this study, *x*^2^*/df* = 2.995, RMSEA = 0.096, GFI = 0.849, AGFI = 0.801, and NFI = 0.856, TLI = 0.880, CFI = 0.898, IFI = 0.899, and RFI = 0.831. All of these indicators meet the recommended criteria, demonstrating that the model has good fit.

### Path Analysis

**FIGURE 2:**
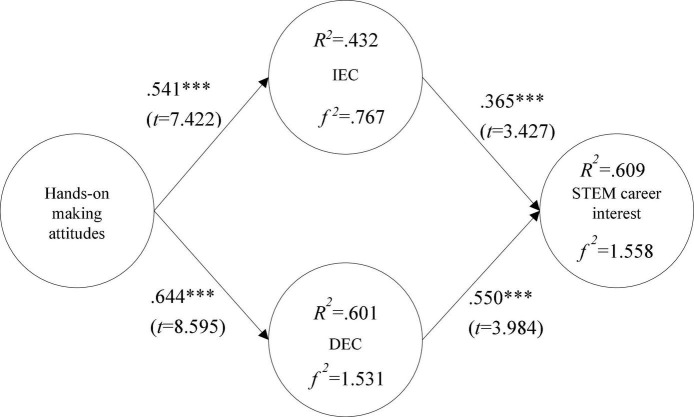
Model fit analysis. ***p < 0.001; **p < 0.01; *p < 0.05.

This study adopted the covariance-based structural equation model. The significance of the paths is determined by the value of each path coefficient (Hair et al., 2019b). Figure 2 shows the validation of the path analysis between hypotheses. H1: the influence of hands-on making attitude on I-type EC was supported with a standardized regression coefficient (SRC) of 0.541 (*t* = 7.422^***^, *p* < 0.001); H2: the influence of hands-on making attitude on D-type EC was supported with a standardized regression coefficient (SRC) of 0.644 (*t* = 8.595^***^, *p* < 0.001); H3: the influence of I-type EC on Students’ STEM career interest was supported with a standardized regression coefficient (SRC) of 0.365 (*t* = 3.427^***^, *p* < 0.001); H4: the influence of D-type EC on Students’ STEM career interest was supported with a standardized regression coefficient (SRC) of 0.550 (*t* = 3.984^***^, *p* < 0.001).

In the path analysis, R-squared (*R*^2^) shows the proportion of variation in the variables explained by the effects of other variables within the model, based on the square of the multiple correlation coefficient (Byrne, 2001). Therefore, when the value of *R*^2^ is closer to 1, the model’s explanatory ability is more powerful. It has been suggested that *R*^2^ larger than 0.67 means the model has good explanatory ability, when it is around 0.33, the model has fair explanatory ability, and when it is around 0.19, it has poor explanatory ability (Awang et al., 2015). The variance of the hands-on making attitude to I-type EC was 43.2%, and to D-type EC it was 60.1%; and for variance of hands-on making attitude, I-type EC and D-type EC to STEM career interest was 60.9%. Those variances were above the suggested threshold value of 10% (Falk and Miller, 1992), indicating that all variables had good predictive power.

The Cohen’s *f*^2^ effect size is defined as follows (Cohen, 1988): Where *R*^2^ is the squared multiple correlation, *f*^2^ ≥ 0.02, *f*^2^ ≥ 0.15, and *f*^2^ ≥ 0.35 represent small, medium, and large effect sizes, respectively. These data can help determine statistical significance, but if confirmed as practically significant, it can be judged according to the verification of the effect quantity. As for the effect sizes in this study, results indicated that hands-on making attitude to I-type EC had a large effect size (*f*^2^ = 0.767); hands-on making attitude to D-type EC had a large effect size (*f*^2^ = 1.531); and hands-on making attitude, I-type EC, and D-type EC to STEM career interest also had a large effect size (*f*^2^ = 1.558).

### Indirect Effect Analysis

The study used Bootstrapping, which means using a small initial program to load the program into the operating system to analyze the indirect effects of this model. When the indirect effects were analyzed, the interval between the two values did not include zero. According to Preacher and Hayes (2008), this shows that the model had an indirect effect. The indirect effect of hands-on attitude on STEM career interest ranged from 0.375 to 0.974. As shown in Table 4, this suggests that there is an indirect effect in this research model (MacKinnon, 2008). As hypothesized, higher levels of hands-on attitude were positively associated with STEM career interest, thus supporting H5. That is, hands-on attitude positively predicted STEM career intentions under the mediation of the two types of EC.

**TABLE 4:** Indirect effect analysis.

	Hands-on making attitude	
	
Construct indirect effect	β	95%CI
STEM career interest	0.627***	[0.375, 0.974]

***The mean difference is significant at the 0.001 level.

## Discussion

The IEYI competition activities provide a learning environment which creates an environment for youth learners to experience the objects of real-world STEM-related tools to solve problems, manipulate the real technologies within the world of work, and experience the complete action process (Hu et al., 2020). Drawing on ABO, this study chose young inventors who participated in an international youth invention exhibition as subjects. How the role of Students’ hands-on making attitude as antecedent to motivate the epistemic behavior to advance STEM knowledge and skills for IEYI contests that explicitly predict Student’s future pursing in STEM-related career. These proposed hypotheses were verified as follows.

The existence of attitudes implicitly impacts on individuals’ behavior, and individuals often refer attitude to the tendency to drive actions (Serenko, 2022). In hands-on making contests, to create functions and ensure the quality of an invention in which that Students’ curiosity can be activated (Mohr-Schroeder et al., 2014). In line with this, in IEYI competitions, students need to complete their works and design excellent products to win the competition. In this process, they have to consistently improve the quality and function of their products; thereby hands-on making attitudes contribute to salient beliefs and outcome expectations (Sukhu et al., 2019). How hands-on making attitudes relate to triggering students’ epistemic curiosity in project design for IEYI was hypothesized, and H1 and H2 were both positively verified. This evidence supported the link between curiosity and attitude toward hands-on making, and is consistent with a previous study which revealed a positive correlation when students were involved in making project with a high degree of uncertainty, novelty preference, and dynamic complexity (Jirout and Klahr, 2012). Moreover, the results are supported by other studies which revealed that a positive hands-on attitude contributed to the development of STEM knowledge and skills when searching for or thinking about solutions to problems (Christensen et al., 2015; Sarı et al., in press).

Individuals’ epistemic curiosity increases their confidence in the STEM field. We drew on the broaden-and-build psychology of working theories to better understand how optimism about one’s career develops and influences one’s vocational interest (Eva et al., 2020). Inquisitive curiosity captures flexibility and risk taking, and optimizes career confidence (Santilli et al., 2017). Careers have been defined as a sequence of work experiences which evolve over a person’s lifetime, and as the movement of a person through time and work space (De Vos et al., 2021). Work experiences help individuals to self-evaluate their person-vocation fit, which in turn builds their personal career confidence and interest (Glosenberg et al., 2019). In line with this, how the efforts of attending IEYI with project making in relation to individuals’ STEM career interest, we applied the broaden-and-build theory to explain. Accordingly, we hypothesized that the two types of EC can predict STEM career interest, and H3 and H4 were positively verified. The results are supported by a previous study; for example, the extent to which interest increases as a consequence of out-of-school program participation is a positive yet trivial probability (Lewalter et al., 2021). Taken IEYI contest as out-of-school activity in an invention-oriented STEM competition, which bring out students have to thinking outside the box in relation to practicing their epistemic curiosity in which their STEM career interests promoted.

With more students eager to participate in STEM technology competitions, a previous study directly spotlighted that Students’ positive attitudes toward STEM practices during competitions can enhance their interest in making STEM projects (Ku et al., 2022). In line with this, out-of-school STEM programs were found to help boost high school STEM career aspirations (e.g., Constan and Spicer, 2015; Kitchen et al., 2018). H5 was positively supported by this research and further explains the reasons. In the process of producing projects, students make alternative models which generates their new perspectives on how they might “do” (STEM) differently (Yanez et al., 2019). IEYI is an integrated STEM contest which provides students with experience of analyzing, designing, verifying, and practicing in hands-on actions to assemble interrelated elements into a functional whole to showcase their competencies (Kim and Kim, 2021). In line with this, hands-on making attitude, as an observed variable in Students’ subjective perceptions of the value of the STEM competition, was shown to have an indirect effect on their STEM career interest. This study contributes to the STEM theory by examining an “attitude-behavior-outcome” framework of STEM hands-on making relationships and distinguishing the influence between IEC and DEC which were activated through different stimulations on Students’ STEM career interest.

## Conclusion

STEM is important because it integrates multiple disciplines, emphasizes learning by doing and experiential learning, and adopts practical skills assessment practices. The aim of STEM education is to provide a seamless gateway for students moving from school to the workplace and to contribute to the increase in Students’ curiosity about the physical world (Jirout and Klahr, 2012) and innovation in their future career development. IEYI is a STEM competition which highlights Students’ learning in science, engineering, mathematics, arts, and technology investigations. In summary, those participants who had more positive hands-on attitudes toward solving little problems in their daily life had higher positive predictive power for IEC and DEC, and both types of EC positively predicted students’ STEM career interest.

### Implications

This study was conducted during a youth invention competition, and the results showed that STEM competitions can be seen as a suitable channel for fostering creative engagement and access to knowledge objects as a way to stimulate Students’ developing interest in STEM. According to Knorr Cetina (2001), “epistemic practice” exists in advanced project design, while Guile (2009) argued that “the accumulation, verification and distribution of knowledge to improve quality is becoming a constitutive feature of innovation.” From this, epistemic curiosity generated from and resting on practice could foster STEM inquiry or reflection on problem solving during the invention process. When attending an invention competition, if students can maintain high levels of EC, the transition from education to work will be supported and more of the twenty-first century workforce will be willing to choose STEM careers in the future. In this case, these findings provide practical guidance as to how educators can be involved with activity-centered STEM outreach design: implicitly designate vocational exploration practices with cognitive challenges for young makers. Activating curiosity as a unique aspiration has important implications for supporting Students’ development as hands-on making inventors.

Using ABO to guide this study was feasible; hands-on making attitudes as an antecedent could activate participants’ epistemic curiosity in the forms of desire to know and need to know. ABO is suitable for analyzing the predictions between perceived behavior and career decision-making after engaging in STEM activities, derived mainly from the interaction between contextual factors such as successful problem solving in the invention process (Christiansen and Tett, 2013), as a way to enhance individuals’ career interest with person-vocation fit. This study suggests that the hands-on making attitude of vocational high school students involved in STEM projects should be improved to initiate their epistemic curiosity and cultivate their long-term career interest in STEM.

### Limitations and Future Study

There are some limitations to the present study that should be noted. Firstly, some researchers have argued that students have already established high levels of STEM interest and positive attitudes prior to outreach program participation (Bachman et al., 2008). Future research can administer pre- and post-program surveys so as to infer the causal effect of STEM competition activities on career intentions.

The mind-sponge mechanism assumes that individuals have a mindset, or a set of core values, which serves as a benchmark for information absorption and multiple filtering (Vuong and Napier, 2015), which has a further positive influence on their thinking and behaviors (Vuong et al., 2022). In that case, when facing a problem that they cannot solve based solely on prior knowledge and skills, Students’ mind-sponge regulates seeking information from external sources to arouse epistemic curiosity. Such epistemic curiosity later appears in the mindset and subsequently influences the subjective cost-benefit judgment during information absorption. Thus, future studies may focus on how the mind-sponge mechanism could be revealed more deeply from epistemic curiosity.

This study was based on an IEYI competition which involved making a product by applying STEM; we therefore put more emphasis on attitude toward career interest when students were engaged in the creative activities. The point of view is from IEYI participants’ perception; however, the scheme of organizing an invention exhibition can influence the willingness of students to endeavor to practice STEM knowledge and skills. This is observed from the enrollment numbers of joining the Taiwan IEYI competition in which the value evaluated by participants affect them to decide how much they will engage in that will develop their STEM interest in that career. This perspective was not included in this study; thus, future studies can focus on the value the rationalize participants career interest.

Finally, we described students’ STEM-related career interest in general, but made no differentiation between STEM career categories, such as science (Biological/Chemical investigators), engineering (e.g., ICT professionals, technicians, and construction workers), medicine (e.g., veterinarians, dosimetrists), or health (e.g., nursing), with no breakdown of Students’ career interest being tracked. It is therefore suggested that future studies can adopt a more fine-grained analysis of Students’ STEM-related domain-specific career interest to better understand whether there are differences according to different categories of STEM.

## Data Availability Statement

The original contributions presented in the study are included in the article/supplementary material, further inquiries can be directed to the corresponding authors.

## Ethics Statement

Ethical review and approval was not required for the study on human participants in accordance with the local legislation and institutional requirements. The participants provided their written informed consent to participate in this study.

## Author Contributions

J-CH, YC, and C-RT: concept and design. C-RT and J-HY: acquisition of data. YC: drafting of the manuscript. J-CH and YC: critical revision of the manuscript. J-HY and YC: statistical analysis. All authors contributed to the article and approved the submitted version.

## Conflict of Interest

The authors declare that the research was conducted in the absence of any commercial or financial relationships that could be construed as a potential conflict of interest.

## Publisher’s Note

All claims expressed in this article are solely those of the authors and do not necessarily represent those of their affiliated organizations, or those of the publisher, the editors and the reviewers. Any product that may be evaluated in this article, or claim that may be made by its manufacturer, is not guaranteed or endorsed by the publisher.
